# Twelve-Month Antiretroviral Therapy Suppresses Plasma and Genital Viral Loads but Fails to Alter Genital Levels of Cytokines, in a Cohort of HIV-Infected Rwandan Women

**DOI:** 10.1371/journal.pone.0127201

**Published:** 2015-05-26

**Authors:** Pascale Ondoa, Raju Gautam, John Rusine, Rene Lutter, Suzanne Jurriaans, Neeltje Kootstra, Etienne Karita, Janneke van de Wijgert

**Affiliations:** 1 Amsterdam Institute of Global Health and Development (AIGHD), Department of Global Health of the Academic Medical Center, Amsterdam, The Netherlands; 2 Institute of Infection and Global Health, University of Liverpool, Liverpool, United Kingdom; 3 INTERACT Program, Kigali, Rwanda; 4 National Reference Laboratory, Kigali, Rwanda; 5 Department of Respiratory Medicine of the Academic Medical Center, Amsterdam, The Netherlands; 6 Department of Experimental Immunology of the Academic Medical Center, Amsterdam, The Netherlands; 7 Department of Medical Microbiology of the Academic Medical Center, Amsterdam, The Netherlands; 8 Project San Francisco, Kigali, Rwanda; Rush University, UNITED STATES

## Abstract

**Background:**

Genital viral load (GVL) is the main determinant of sexual transmission of human immune-deficiency virus (HIV). The effect of antiretroviral therapy (ART) on local cervico-vaginal immunological factors associated with GVL is poorly described. We aimed to identify the risk factors of detectable GVL, and the impact of ART on HIV genital shedding and its correlates in a cohort of HIV-infected women, attending HIV care in Kigali, Rwanda.

**Materials and Methods:**

All participants were evaluated for GVL, plasma viral load (PVL), CD4 count, various sexually-transmitted infections (STIs) at baseline and at month 12. Genital concentration of 19 cytokines and mRNA expression of APOBEC3G and BST2, two host HIV restriction factors, were evaluated at baseline in all participants. Cytokine levels were re-assessed at month 12 only in participants eligible for ART at baseline. Risk factors of GVL ≥40copies/mL at baseline and month 12 were assessed using logistic regression. Effect of 12-month ART on various local and systemic immunological parameters was examined using a paired t-test and McNemar as appropriate.

**Results:**

96 of the 247 women enrolled in the study were eligible for ART. After 12 months of ART, PVL and GVL decreased to undetectable level in respectively 74 and 88% of treated participants. ART did not affect cytokine levels. HIV genital shedding occurred only when PVL was detectable. At baseline, GVL was independently associated with IL-1β after controlling for PVL, age and *N*. *gonorrhea* infection (95% CI 1.32-2.15) and at month 12 with MIP-1β (95% CI 0.96-21.32) after controlling for baseline GVL, PVL and month 12 IL-8.

**Conclusion:**

Suppressive ART does not necessarily reduce genital level of immune activation. Minimizing all conditions favoring genital inflammation, including active detection and treatment of STIs, might reduce the risk of HIV transmission as supplement to the provision of potent ART.

## Introduction

Sub-Saharan Africa bears 70% of the worldwide prevalence of HIV infections, with the majority of new infections occurring in women [1]. The concentration of HIV RNA in cervico-vaginal fluids (referred to as the genital viral load (GVL) or genital shedding) is an important determinant of HIV transmission from HIV-infected women to their unborn children and neonates as well as their sexual partners [2]. HIV genital shedding has been demonstrated to be primarily associated with the HIV RNA concentration in plasma (referred to as the plasma viral load (PVL)) [3–5] but also to depend on various other general or local factors [6]. These factors include, among others, local inflammation (for example, inflammation caused by sexually transmitted co-infections), hormonal changes associated with the menstrual cycle, pregnancy or contraception, and peripheral or mucosal CD4 count [7–12]. Genital infections are known to increase the odds of HIV genital shedding but the mechanisms are incompletely understood [13]. Inflammatory cytokines and chemokines secreted during or outside episodes of genital infections are proposed to induce HIV replication directly by promoting viral transcription of infected cells [14], to favor viral transudation through increased vascular permeability or disruption of the mucosal barrier [15] or to stimulate the recruitment of target cells on the site of inflammation [16].

Conversely, cytokines such as IFN-α and IP-10 might also reduce HIV replication through the regulation of the cellular expression of intrinsic anti-viral factor APOBEC3G and tetherin (BST2). Host restriction factors have been demonstrated to inhibit HIV-1 replication *in vitro* and therefore represent promising candidates for innate immune-based therapeutic strategies [17, 18]. APOBEC 3G is part of the APOBEC family and interferes with proper HIV replication by inducing G-to-A hypermutations in the viral genome. The effect of APOBEC proteins are counteracted by the HIV-1 gene product. Viral infectivity protein (*Vif*), which triggers the ubiquitination and degradation of APOBEC3G via the proteasomal pathway [19]. BST2 impairs the release of nascent virions by tethering them on the surface of infected cells [20]. BST-2 restriction is counteracted by the HIV-1 gene product, 16-kDa viral protein U (*Vpu*) from infected cells. APOBEC3G and BST2 are expressed by immune cells from various tissues, including the vaginal mucosa. Whether the local expression of these innate viral restriction factors plays a role in the modulating effect of cytokines and vaginal pathogens on HIV genital shedding has never been studied.

PVL suppression to undetectable level is the hallmark of potent antiretroviral therapy (ART) However, ART-induced virological suppression in the plasma does not always correlate with a GVL below the level of detection [21–23], representing an enduring risk for HIV transmission. Despite the reduction of PVL to undetectable concentrations, ART usually fails at completely normalizing systemic immune activation [24, 25]. The interaction between HIV therapy, HIV genital shedding and markers of local immune activation, including cytokines, is being less well studied and not completely clear. These data can support the design of adequate interventions to eliminate the risk of sexual HIV transmission.

We investigated the factors associated with having a detectable GVL as well as the impact of ART on GVL and genital inflammation, in a cohort of HIV-infected women in Rwanda.

## Materials and Methods

The SEARCH study has been described previously [26, 27] and was conducted from November 2007 until August 2010 at the outpatient clinic of the center for treatment and research on AIDS, tuberculosis and malaria (TRAC-Plus), which is now part of the Institute of HIV/AIDS, Disease prevention and control (IHDPC) within the Rwandan Biomedical Center.

### Study participants and design

The SEARCH study was designed to enroll at least 100 HIV-infected women immediately eligible for ART according to the Rwandan national guidelines [28] (ART group) and 200 (pre-ART group) women not yet qualifying for ART. The clinical ART eligibility criteria at that time was CD4 count <350 cell/μL or WHO clinical stage IV, regardless of the CD4 count. Other inclusion criteria were being 18 years of age or older, residing and planning to reside within travel distance from the TRAC-Plus clinic for the duration of the follow-up, willing and able to adhere to study protocol, and willing and able to give written informed consent for enrolment in the study. The main exclusion criteria were pregnancy and laboratory or clinical diagnosis or suspicion of tuberculosis. Previous use of antiretroviral drugs in the context of prevention of mother-to-child transmission was not an exclusion criterion.

Participants eligible for treatment were provided with ART through the national HIV treatment program. Participants received a first-line regimen in accordance with the 2007 Rwanda National ART treatment guidelines and in line with WHO recommendations used at that time [28, 29]. When patients from the pre-ART group became eligible for treatment, they were switched to the ART group. First-line regimens included a combination of either two Nucleoside analogue Reverse Transcriptase Inhibitors (NRTI) and one Non-Nucleoside Reverse Transcriptase Inhibitor (NNRTI) or a combination of three NRTI. Participants on ART were routinely monitored on the basis of clinical symptoms and CD4 count as recommended by the national guidelines [28].

The treatment of STIs followed the syndromic management guidelines because laboratory results were not available in real time. Syndromic treatments were not recorded in the frame of this study. Positive herpes serology and positive Human Papilloma Virus (HPV) PCR were not treated; women were given their test results and given advise on 1) how to prevent transmission of *Herpes simplex* type 2 (HSV-2) to partners, and 2) to get regular Pap smears to prevent cervical cancer.

### Clinical procedures

All patients were seen at baseline, and every three months for 6 to 24 months thereafter. Patients initiating ART received additional visits in the first trimester of the follow up, visits at week 2, month 1 and month 2. All patients were interviewed for information on socio-demographics, sexual and contraceptive behavior as well HIV and reproductive health history.

Blood samples were collected at all clinic visits. They were hand-carried from the clinic to the laboratory next door within 4 hours after collection. CD4^+^T cell counts on EDTA blood samples were always done on the day of sample collection. All other blood samples were stored as aliquots of plasma, serum or buffy coats at −80°C until further processing and testing.

Endocervical specimens were collected every 6 months during pelvic exams. A swab was vigorously agitated in Amplicor specimen transport medium (Roche Molecular Systems, Branchburg, NJ, USA), and a cytobrush in PreservCyt medium (Hologic, Bedford, MA, USA).

Cervico-vaginal lavages (CVL) were obtained by irrigating the left and right fornix and cervical os twice using 5mL normal saline. The liquid was subsequently aspirated after 30 seconds. The CVL fluid was immediately placed on ice or at 4 –8°C and centrifuged at 1,000 rpm for 10 min to separate the liquid phase from the cells. The cell pellet was re-suspended in 1 ml of PBS, centrifuged again, and cells were stored at -80°C. Cells pellets and aliquots of supernatant were stored at -80°C until further processing.

### Laboratory procedures

GVL, cytokine, and APOBEC3G and BST2 gene expression testing was done at the Academic Medical Centrum (AMC) in Amsterdam, the Netherlands. All other laboratory tests were carried out at the National Reference Laboratory in Kigali, Rwanda,

CD4^+^T cell counts (FACSCalibur, Becton Dickinson, San Jose, CA, USA) were measured every 3 months for women who did not yet qualify for ART and every 6 months for those on ART. PVL testing (COBAS AmpliPrep/COBAS TaqMan HIV-1 Test versions 2.0, Roche Molecular Diagnostics, Pleasanton, CA, USA) was done at ART initiation and every 12 months thereafter. The lower limit of detection was 40 HIV RNA copies/mL. Women were tested for pregnancy using an hCG urine dipstick test at baseline and every 6 months. Participants were tested for *Herpes simplex* type 2 (HSV-2) using HerpeSelect test kits (Focus Diagnostics, Cypress, CA, USA) at baseline, for syphilis by RPR confirmed by TPHA (Human Diagnostics, Wiesbaden, Germany) at baseline and every 6 months, and for *Neisseria gonorrhea* and *Chlamydia trachomatis* by PCR (COBAS Amplicor, Roche Molecular Systems, Branchburg, NJ,USA) at baseline and every 12 months.

CVL supernatants were shipped to the AMC in Amsterdam on dry ice, thawed, and 500μL of CVL was mixed to 500μL of phosphate buffer saline. The GVL was determined by nucleic acid amplification using COBAS/Ampliprep/COBAS Taqman v2.0 according to the manufacturer instructions (Roche Molecular Systems, Branchburg, NJ, USA). Quantification of cytokines in CVLs was done on diluted samples (4x) using a Luminex-based multiplex system (Bio-Plex Human Cytokine 27-plex panel, Bio-Rad Laboratories, Hercules, CA, USA) per manufacturer’s instructions. Nineteen pro- (TNF-α, IL-1α, IL-1β, IL-6, IL-12_p70_, IL-17, IFN-α,), anti-inflammatory cytokines (IL-10, IL-1RA,), chemokines (IL-8, IP-10, MCP-1, MIP-1α, MIP-1β, RANTES), adaptive immune mediators (IL-2, IFN-γ) and growth factors (VEGF, G-CSF), were selected based on their potential involvement in the inflammation process of the genital tract. Each standard curve was fitted using Bio-Plex manager 6.0 software and was based on 11 standards (8 recommended plus 3 higher dilutions of the standards).

The lowest point of each calibration curve was regarded as the lower limit of reliable detection. Genital levels of cytokines were re-evaluated at month 12 only in participants receiving ART.

Expression of APOBEC3G and BST2 mRNA in CVLs and blood cells was measured in baseline samples using RT-qPCR. RNA was isolated from the CVL cell pellets or buffy coats using TriPure Isolation Reagent (Roche). The concentration of the isolated RNA was measured on a nanodrop (ND1000 Isogen Lifescience). cDNA was prepared from 500ng of RNA using the Transcription RT Reaction Buffer as recommended by the manufacturer (Roche Transcriptor First Strand cDNA Synthesis Kit). The qPCR was performed with a Lightcycler 480 using specific primer pairs for APOBEC3G and BST2 [30] and SYBR Green I Master (Roche). Two μL of cDNA were used in the qPCR reaction, with the following cycling conditions: denaturation: 95°C for 10 min; amplification: 50 cycles at 95°C for 10 sec, 58°C for 20sec and 72°C for 30 sec. Purity of the PCR products was confirmed by melting curve analysis. β-actin expression levels were used to correct for cDNA input. A serial dilution of the 8E5 cell DNA was used as a standard curve for β-actin [31].

### Ethical considerations

Ethical approval was obtained from the Rwandan National Ethics Committee. All study participants provided written informed consent prior to enrollment, were free to withdraw from the study at any time, and were transferred to publicly-funded HIV treatment programs at the end of their study participation. Participants with curable genital infections were treated according to the national treatment guidelines.

### Statistical analysis

The PVL and GVL data were dichotomized based on the lower detection limit (< 40 copies/ml) of the viral load assay. Individuals with < 40 copies/ml of virus were categorized as having low (non-detectable) level of virus and those with ≥ 40 copies/ml of virus as having high (detectable) level of virus.

Cytokines concentrations above the highest and below the lowest cytokine concentration of each respective standard curve were identified as out of range (OOR) values. Cytokines for which < 15% of the readings were OOR values were analyzed as continuous variables. Cytokines giving readings between 15 and 50% OOR values were analyzed as binary variables (present/absent). Cytokines with more than 50% OOR read-out values were removed from the analysis. For cytokines that qualified for analysis, the OOR values below the lowest point of standard curve were assigned a value of one half of the lowest concentration of the standard curve and OOR values above the highest point of the standard curve were assigned a value of 1.5 times the highest concentration of the standard curve of the respective cytokines. The concentrations of APOBEC3G, BST2 and cytokines qualifying for analysis as continuous variables were log (base 10) transformed.

All binary and categorical variables were summarized as counts (proportions) and continuous variables as mean (standard deviation, range). Differences at baseline between low and high GVL groups were assessed by t-test for continuous variables and Fisher’s exact test for proportions. Logistic regression was used to assess factors associated with GVL at baseline with GVL categorized as absent (undetectable) or present (detectable). In the multivariable model, all variables with P value < 0.2 in the bivariable analyses were considered, and model selection was performed using stepwise backward elimination method. The final model was selected using the Akaike Information Criteria (AIC). Similar analyses were performed to determine the effect of ART on GVL (present/absent) upon 12 months of treatment after controlling for the baseline level of genital viral load and other factors. For the final model, assumption of normality was assessed using residual plots and linearity for continuous variables was assessed by plotting log odds of predicted values against the continuous variables. All statistical analyses were performed using R version 3.1.1 (The R Project for Statistical Computing, http://www.r-project.org/).

## Results

### Cohort profile

**Fig 1:**
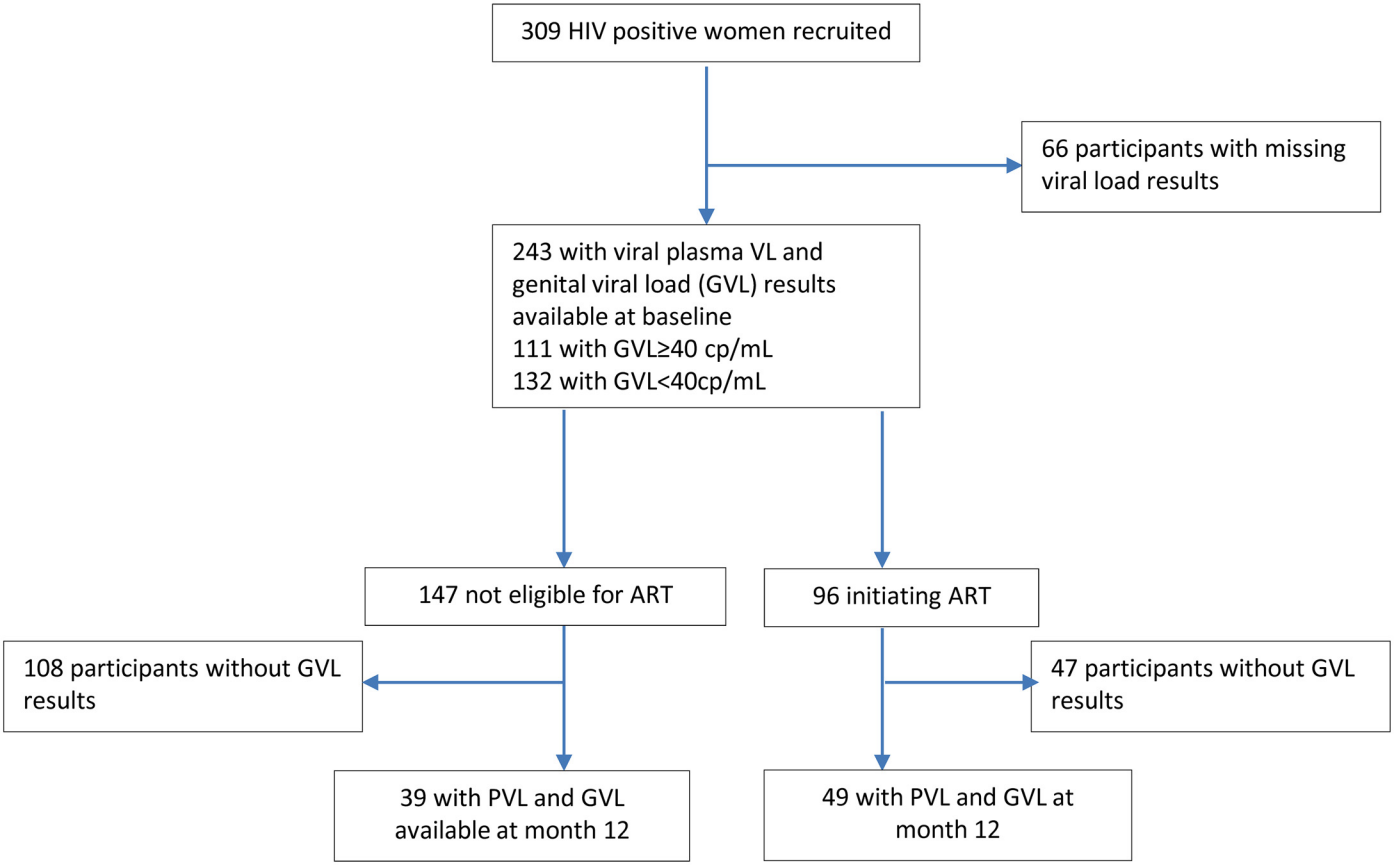
Cohort profile. This flow chart provides information on the number of patients recruited, eligible for antiretroviral therapy and who had viral load and CD4 data available at baseline and at month 12. Patients were classified in groups of genital viral load (GVL) <40 RNA copies/ml and GVL ≥40 copies /mL.

Three hundred and nine women were recruited in the study at baseline. Of these, 243 had PVL and GVL results available at baseline (Fig 1, S1 Dataset): 132 of them were categorized as GVL undetectable (<40 HIV RNA copies/mL) and 111 as GVL detectable (≥40 HIV RNA copies/mL). Ninety six of the 243 participants were initiated on ART while the remaining 147 were not yet eligible for therapy. All participants were followed for 12 months but only 49 in the ART group and 39 in the pre-ART group had PVL and GVL data available at month 12. A variable number of participants had measurements available for genital cytokines, APOBEC3G and BST2 for the two time points (see table and figure legends).

### Characteristics of the study population

Participants with detectable versus undetectable GVL at baseline were similar in terms of education levels, social status and utilization of family planning methods (Table 1). Women with undetectable GVL were slightly younger than the other study participants (mean age = 32.73 versus 35.52 years, p = 0.009). *Herpes simplex* type 2 infection was very common in both groups (85% and 93%; p = 0.100). Other STIs were less frequent, with only *Neisseria gonorrhea* being significantly more frequent in women with detectable GVL (5% versus 16% of participants, p = 0.027). Although the two groups did not differ in terms of clinical stage of HIV disease (p = 0.306), CD4 count and PVL levels reflected a more advanced disease progression in the group with detectable GVL compared to the group with undetectable GVL (mean CD4 count is log_10_ 2.46 versus 2.63 cells/μL, respectively (p = 0.0001) and PVL is log_10_ 4.51 versus 3.53 RNA copies/mL, respectively (p < 0.0001)).

**Table 1:** Characteristics of the study population.

Variables	N	Genital HIV RNA VL <40 copies /mL	Genital HIV RNA VL ≥40 copies/mL	P value
		N = 132	N = 111	
**Age^#^ (mean years, SD)**	238	32.73 (7.76)	35.52 (8.44)	0.0089*
**Education level (N,%)**	237			
** None**		14 (11)	9 (8)	0.3602
** Post-secondary**		3 (2)	7 (6)	
** Primary school or less**		70 (55)	54 (5)	
** Secondary school or less**		41 (32)	39 (36)	
**Pelvic abnormality detected (N, %)**	243	89 (67)	73 (66)	0.7866
**Family planning method (N, %)**	240			
** None**		95 (73)	92 (84)	0.2142
** Pill**		7 (5)	3 (3)	
** Injectable**		20 (15)	11 (1)	
** Implants**		7 (5)	2 (2)	
** Intra Uterine device**		1 (1)	2 (2)	
**Use of any female contraception method (N,%)**	240	35 (27)	18 (16)	0.0608
**Condom use during the last sex act (N,%)**	235	57 (44)	44 (42)	0.6934
**WHO disease stage class (N,%)**	233			
** I**		97 (76)	78 (74)	0.3609
** II**		23 (18)	16 (15)	
** III**		6 (5)	11 (1)	
** IV**		1 (1)	1 (1)	
**Marital status (N,%)**				
** Married**	240	74 (57)	56 (51)	0.6444
** Divorced**		19 (15)	18 (16)	
** Widowed**		30 (23)	26 (24)	
** Single**		7 (5)	10 (9)	
***N*. *gonorrhea* infection (N,%)**	188	5 (5)	14 (16)	0.027*
***C*. *trachomatis* infection (N,%)**	188	1 (1)	4 (4)	0.1913
***H*. *simplex* type 2 infection (N,%)**	239	111 (85)	101 (93)	0.1004
**Syphilis infection (N,%)**	238	3 (2)	5 (5)	0.475
***T vaginalis* infection (N,%)**	184	9 (9)	9 (11)	0.8048
**Any concomitant STI (N,%)**	222	111 (94)	102 (98)	0.1787
**Genital HIV RNA [Log_10_ copies/mL (mean, SD; min-max)]**	243	1.3 (0; 1.3–1.3)	3.24 (0.95; 2.64–6.48)	0*
**Plasma HIV RNA [Log_10_ copies/mL (mean, SD; min-max)]**	239	3.53 (1.13; 1.3–5.96)	4.51 (0.88; 2.64–6.48)	0*
**CD4 count [Log_10_ cells/μL (mean, SD; min-max)]**	243	2.63 (0.34; 0.3–3.88)	2.46 (0.29;1.54–3.06)	0.0001*
**Genital APOBEC3G [Log_10_ copies/10^6^ cells (mean, SD; min-max)]**	93	4.47 (0.67; 2.6–5.72)	4.5 (0.71; 3.1–6.09)	0.8136
**Genital BST2 [(Log_10_ copies/10^6^ cells (mean, SD; min-max)]**	35	1.95 (0.8; 0.48–3.39)	1.4 (0.62; 0–2.21)	0.0315*
**APOBEC3G in PBMC [(Log_10_ copies/10^6^ cells (mean, SD; min-max)]**	61	5.03 (0.84; 3.76–6.82)	5 (0.9; 3.52–7.29)	0.9284
**BST2 in PBMCs [(Log_10_ copies/10^6^ cells (mean, SD; min-max)]**	61	0.09 (2.11; -2.77–2.57)	0.36 (-3.4–2.86)	0.621
**IL-8 [log_10_ pg/mL (mean, SD; min-max)]**	225	2.49 (0.91; 1.3–4.41)	3 (0.95; 1.3–4.89)	0.0001*
**IP-10 [log_10_ pg/mL (mean, SD; min-max)]**	225	3.06 (1.07; 0.58–6.34)	3.33 (1.07; 0.58–5.66)	0.0584
**MIP-1 β [log_10_ pg/mL (mean, SD; min-max)]**	225	-0.04 (0.64; -0.8–2.28)	0.23 (0.83; 1.18–4.88)	0.0082*
**VEGF [log_10_ pg/mL (mean, SD; min-max)]**	225	2.88 (0.86; 1.18–4.91)	3.21 (0.88; 2–3.78)	0.0047*
**IL-1β [log_10_ pg/mL (mean, SD; min-max)]**	225	-0.02 (1.56; 2–3.78)	1.03 (1.52; 2–2.44)	0*
**Detectable IL-1RA (N, %)**	225	121 (99)	102 (99)	1
**Detectable IL-6 (N, %)**	225	57 (47)	61 (59)	0.0813
**Detectable G-CSF (N, %)**	225	83 (68)	87 (84)	0.005*
**Detectable MCP-1 (N, %)**	225	77 (63)	70 (68)	0.4838
**Detectable IL-1α (N, %)**	225	72 (59)	68 (66)	0.3342

Groups of participants with genital viral load (GVL) < 40 HIV RNA copies/mL (N = 132) versus GVL≥40 HIV RNA copies/mL (N = 111) at baseline are compared. Comparison is made using t-test for continuous and Chi-square for dichotomous variables. Significant differences are highlighted with*.

### Comparison of CVL cytokine levels in participants with detectable versus undetectable GVL at baseline

Cytokine concentrations in CVLs were measured in a total of 225 participants at baseline (Table 1). Nine cytokines (IL-2, IL-10, IL-12_p70_, IL-17, IFN-γ, MIP-1α, RANTES, TNF-α and IFN-α) had more than 50% OOR values and were removed from the analysis (S2 Dataset). The detection range of the 10 cytokines qualifying for the analysis varied between 4.07 and 3.67 Log_10_ pg/mL for the highest concentrations and between 1.17 and 2.79 Log_10_ pg/mL for the lowest concentrations (S2 Dataset). Five cytokines (IL-1RA, IL-6, G-CSF, MCP-1 and IL-1α) qualified for analysis as binary variables and 5 cytokines (IL-1β, IL-8, IP-10, MIP-1β and VEGF) were analyzed as continuous variables. Levels of IL-8, MIP-1β, VEGF, IL-1β and GCSF were significantly higher in participants with detectable GVL as compared to participants with undetectable GVL (Table 1, S1 Dataset).

### APOBEC 3G and BST-2 expression and HIV RNA load in genital and blood compartments

mRNA expression of APOBEC3G and BST2 were respectively measured in 35 genital cell pellets and 61 PBMC samples at baseline. At baseline, levels of genital expression of BST-2, but not APOBEC3G were significantly lower in women with undetectable GVL as compared to women with detectable GVL (log_10_ 1.95 versus log_10_ 1.4 mRNA copies/10^6^ cells, p = 0.0315, Table 1, S1 Dataset). In contrast, expression of APOBEC3G and BST-2 in PBMCs were not correlated with PVL levels at baseline (data not shown).

BST2 and APOBEC3G expression were positively correlated in the genital tract (Pearson R = 0.6, p = 0.0011), but negatively correlated in the blood compartment (Pearson R = -0.8, p<0.000, data not shown).

The relationships between IFN-α and IFN-γ levels and the genital expression of HIV restriction factors could not be investigated due low levels of interferons in CVL supernatants. The concentration of soluble genital IP-10, which is induced by interferons, was not significantly correlated to levels of genital BST2 or APOBEC3G mRNA (data not shown). IP-10 levels in CVL supernatant did not differ in samples with high versus low levels of BST2 mRNA (log_10_ 3.37 mRNA copies/10^6^ cells in both groups, p = 0.39) or APOBEC3G mRNA in genital cells (log_10_ 3.46 versus log_10_ 3.21 mRNA copies/10^6^ cells, p = 0.46).

### Effect of 12 month ART on GVL and its correlates

**Fig 2:**
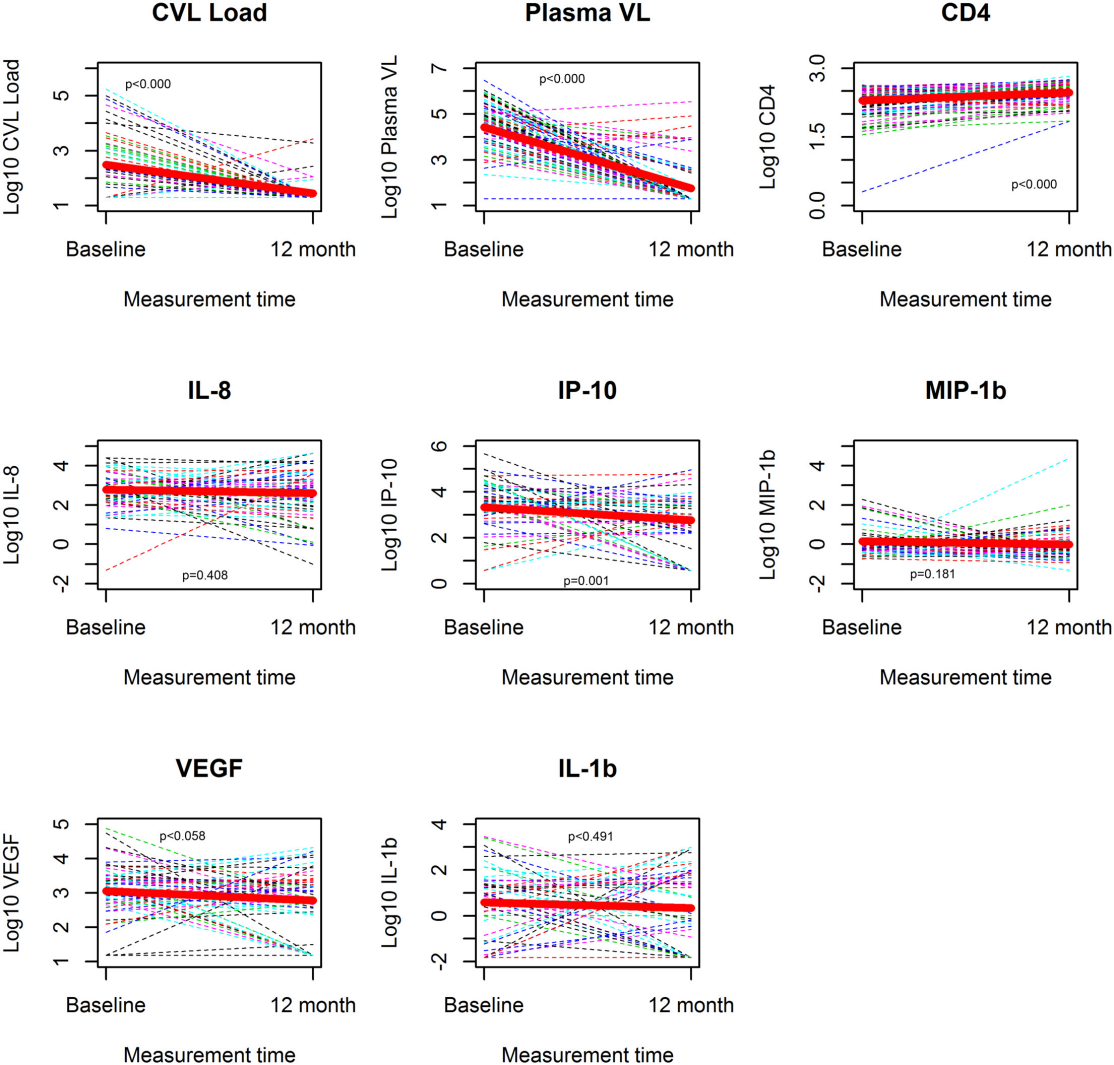
Changes of biological marker levels in the genital compartments between baseline and month 12 among study participants receiving anti-retroviral therapy. Changes of genital (GVL) and plasma viral loads (PVL), CD4 counts and cytokines levels analysed as continuous variables were compared between baseline and month 12 in patients receiving antiretroviral therapy. Thin broken lines represent the change in immune parameter of a study participant between the two measurement times and the thick solid line in red is the average change in the parameter between the two time points. P values were calculated using the paired t-test and are displayed inside each graph. GVL, PVL and CD4 counts changes were significant, whereas cytokines levels remained comparable at baseline and month 12.

Forty-nine of the 96 women (51%) that had initiated ART and 38 of 147 (26%) women not eligible for treatment had their genital HIV viral load measured at month 12. Among treated participants, the mean genital viral load decreased from 2.34 log_10_ RNA copies at baseline to 1.44 log_10_ RNA copies/mL at month 12 (p<0.000, Fig 2 and S1 Dataset). In this group, the proportion of women with genital HIV RNA shedding decreased from 55% at baseline to 12% at month 12 (p<0.000, data not shown,). The mean PVL of treated participants decreased from 4.46 log_10_ RNA copies/mL at baseline to 1.76 log_10_ RNA copies/mL (p<0.000, Fig 2 and S1 Dataset) while CD4 count increased from 2.24 log_10_ cells/μL at baseline to 2.43 log_10_ cells/μl (p<0.000, Fig 2 and S1 Dataset). Eight of the 49 participants receiving ART had PVL>1,000 RNA copies/mL at month 12, which is compatible with the WHO-definition of virological failure [32]. The HIV drug resistance outcomes of women failing ART at month 12 have been published elsewhere [26] and did correlate with the presence of HIV RNA genital shedding (data not shown) at month 12. Among women not receiving ART, there were no significant differences in mean GVL, PVL and CD4 count between baseline and month 12 (data not shown).

Cytokine levels remained comparable or decreased between baseline and month 12 for both treated (Fig 2, Table 2 and S1 Dataset) and untreated participants. Among ART naive women, levels of cytokines decreased significantly for MCP (p = 0.005), IL-1α (p = 0.029), IP-10 (p<0.000) and MIP-1β (p = 0.0053, data not shown). In this group viral load parameters increased (from 1.49 to 1.72 log_10_ RNA copy/mL, p = 0.068 for GVL and from 3.5 to 3.58 log_10_ RNA copies/mL, p = 0.444 for PVL, data not shown) while CD4 count significantly decreased from baseline to month 12 (from 2.76 to 2.7 log_10_ CD4 cells/mL, data not shown). Among treated patients, IP-10 was the single cytokine of which average concentration significantly decreased from 3.44 to 2.64 log_10_ pg/mL (p = 0.001) between baseline and month 12. In this group, levels of all other measurable cytokines remained comparable between baseline and month 12 (Fig 2 and Table 2), in contrast to viral load and CD4 count parameters.

**Table 2:** Changes in proportion of detectable cytokine levels from baseline to month 12 in participants receiving ART,

Variables	N	Baseline	12 months ART	P
**Detectable IL-1RA**	51	50 (98%)	49 (96%)	1
**Detectable IL-6**	51	25 (49%)	18(35%)	0.248
**Detectable GCSF**	51	39(76%)	37(72%)	0.502
**Detectable MCP-1**	51	31 (60%)	28(54%)	0.676
**Detectable IL-1α**	51	31 (60%)	23 (45%)	0.153

Only cytokines analyzed as dichotomous variable are included in this table. Changes of proportion of women with detectable cytokines are compared between baseline and month 12 using McNemar. No significant differences were found. Results on cytokines analyzed as continuous variables are shown in Fig 2.

### Correlates of GVL at baseline and at month 12

Based on the results of the bivariable analysis on baseline parameters (Table 1), PVL, CD4 count, age, IL-8, IP-10, MIP-1β, IL-1β, *N*. *gonorrhea*, *C*. *trachomatis* and HSV type 2 were selected and assessed in the multivariable analysis. The most parsimonious model (AIC = 183.62) indicated that risk factors for detectable GVL at baseline were baseline PVL (adjusted OR (aOR) = 2.47 95% CI: 1.66–3.67) IL-1β concentration in CVLs (aOR = 1.68, 95% CI: 1.32–2.15), after adjusting for age and *N*. *gonorrhea* (Table 3). The odds of detectable GVL were on average 4.6 times greater among women with *N*. *gonorrhea* infection than non-infected women, after adjusting for age, PVL and IL-1β concentration.

**Table 3:** Predictors of GVL at baseline and at month 12 among participants receiving ART.

	Variables	adj. OR	95% CI	P-value
**Baseline model**	Plasma viral load	2.47	1.66, 3.67	< 0.0001
**AIC = 183.62 (N = 96)**	Detectable IL-1β	1.68	1.32, 2.15	<0.0001
	Age	1.04	0.99, 1.09	0.11
	*N*. *gonorrhoea* at baseline.	4.6	1.26, 16.82	0.021
**Month 12 model**	Genital viral load at baseline	0.62	(0.12, 3.27)	0.57
**AIC 24.6 (N = 49)**	(Log_10_ RNA copies/mL)			
	IL-8 at baseline (Log_10_ pg/mL)	5.47	(0.61, 49.52)	0.13
	Plasma Viral load at 12 month	2.3	(0.84, 6.3)	0.1
	(Log_10_ RNA copies/mL)			
	MIP-1β at month 12	4.53	(0.96, 21.32)	0.06
	(Log_10_ pg/mL)			

Predictors of genital viral load ≥40 copies/mL at baseline and at month 12 were determined. Ninety six women initiating ART were included in the analysis at baseline. Forty-nine women receiving ART had viral load data available at month 12 and were included in the analysis at month 12. The variables included were:

At baseline: Plasma viral load, CD4, age, IL-8, IP-10, MIP-1b, VEGF, IL-6, GCSF, IL-1β, *N*. *gonorrhoea*, *Chlamydia trachomatis*, *Trichomonas vaginalis*, HSV2

At month 12: Use of family planning method, marital status, *Trichomonas vaginalis* at baseline & month 12, IL-8 at baseline & month 12, IP-10 at baseline & month 12, MIP-1b at month 12, IL-1β at month 12, IL-1RA at month 12, IL-6 at month 12, Genital VL at baseline, plasma VL at month 12.

AIC: Aikaike Information criteria.

Persistently detectable genital viral load at month 12 was measured only in 2 participants receiving ART, while 4 participants that had undetectable levels of GVL at baseline presented detectable levels of GVL after 12 months. The most parsimonious model (AIC = 24.6) consisted of PVL at month 12 (aOR = 2.3; 95% CI: 0.84–6.3) and MIP-1β at month 12 (aOR = 4.53; 95% CI: 0.96–21.32), after controlling for the baseline levels of GVL, PVL and IL-8 (Table 2 and S1 Dataset).

## Discussion

Twelve months of ART successfully reduced PVL and GVL to undetectable levels in the majority of participants but had no effect on genital levels of inflammatory markers. All patients with detectable GVLs also had detectable PVLs. In contrast, no women with undetectable PVLs had detectable GVLs, suggesting that PVL, which is an essential marker of disease progression is the most important determinant of GVL [2–4]. GVL was also independently associated with local factors including cytokine level in CVLs and concurrent *N*. *Gonorrhea* infection.

Although the proportion of detectable HIV genital shedding decreased substantially after one year ART, it remained strongly associated with detectable PVL at month 12. These observations contrast with previous findings reporting HIV genital shedding in up to a third of women with undetectable PVL [25, 26]. It is possible that some blips of HIV genital shedding were missed in participants with undetectable PVL, due to the wide follow-up interval used in our study or that adherence to ART was particularly high in this setting.

Interestingly, the highly effective ART had no effect on cytokine levels in genital secretion. Cytokine levels remained largely unchanged at month 12, showing even less variation as compared to the untreated group. Much of the inflammation in the genital tract may not be primarily related to GVL. The decrease of some cytokine levels observed in both treated and non-treated participants at month 12, could relate to the syndromic treatment of STIs during routine HIV care. Interestingly, the significant reduction of IP-10 concentrations among treated participants at month 12, suggest that ART had a significant reduction effect only on anti viral IFN-(induced) responses. For the other cytokines evaluated, treatment-induced changes might have been be masked by genital inflammation caused, for instance by concurrent STIs. The possible role of non viral infections in fueling mucosal inflammation is corroborated by findings from a similar study conducted in India [33], whereby levels of genital cytokines significantly decreased after ART in participants tested negative for genital infection and cervical dysplasia. Interestingly, Mkhize and colleagues reported that ART significantly decreases plasma and genital VL but does not alter IL-6 and IL-1β in South African participants with no clinical signs of STI but who were not confirmed free from genital infection through laboratory testing [34].

Our findings have the following implications. First, they corroborate the hypothesis that providing effective highly active ART reduces the risk for sexual HIV transmission [35]. Secondly, since genital inflammation can promote HIV replication on its own, the unaltered profile of cytokine genital secretion upon suppressive ART, represents an enduring, albeit small risk for HIV genital shedding. Persistent genital inflammation despite ART could be related to chronic bacterial vaginosis [8], reported as relatively frequent in this particular setting and likely to be left untreated because most women usually remain asymptomatic. This possibility could not be verified, as patients were not assessed for bacterial vaginosis in the scope of this study. Stable cytokine levels despite undetectable GVL may also pertain to inappropriate practices promoting local irritation, inflammation and loss of balance of the vaginal microbiota [36], such as vaginal cleansing [37].

Although residual genital inflammation did not translate into GVL in this particular cohort, our results suggests that treating STIs, reducing local inflammation, combating inappropriate hygiene practices or restoring the balance of the vaginal flora would contribute to the normalization of genital cytokine secretion profile and the risk reduction of HIV genital shedding. Additional studies are warranted to explore the potential benefit of local anti-inflammatory treatment and/or topical administration of commensal bacteria to the genital tract. Whether more proactive detection and treatment of STIs have additional advantages, as compared to the syndromic STI treatment approach currently recommended by the Rwandan ART program [32], need to be determined. Alternatively, persistent genital immune activation in the face of undetectable GVL could be linked to either systemic parasitic, tuberculosis or malaria infections or to the HIV-induced residual systemic inflammation despite ART-suppressed PVL. Current evidence however does not show any associations between systemic and genital levels of immune activation [33, 38].

At baseline, the CVL concentrations of only a few cytokines and growth factors (IL-8, IL-1β, MIP-1α, VGEF and GCSF) were elevated in women with detectable GVL. These findings are in line with previous reports identifying the same immune factors as correlates of GVL in the context of genital infection and/or local immune activation [10].

At baseline levels of IL-1β were still associated with GVL after adjusting for PVL, age and *N*. *gonorrhea* infection, while the effects of IL-8, MIP-1α, VEGF and GCSF were attenuated. Our data suggests that, compared to the other cytokines studied, the secretion of IL-1β might be more closely associated to genital infections not assessed in this cohort or to inflammation related to non-infectious events. Our observations are in line with the identification of IL-1β as a primary pro-inflammatory mediator secreted in the inflammasome in response to various triggers including bacterial as well as viral infections [39]. Notably, IL-1β has been associated with bacterial vaginosis in several studies [40–42] and further explorations are needed to establish the directional link between IL-1β secretion, bacterial vaginosis and HIV genital shedding.

Conversely, a number of cytokines, including IL-6, MCP-1, IL-1α and IL-1RA generally found to be associated with GVL [37] had comparable levels in the group of women with and without detectable HIV genital shedding. It is possible that the lack of difference observed, relates to generalized local inflammation or conditions such as HSV-2 infection, found in more than 80% of all participants, and not directly to detectable GVL.

The precise cytokine profile predicting genital shedding was different between our study and others, on the one hand and between baseline and month 12, on the other hand. This could reflect the multi-factorial causes of HIV genital shedding in the context of a fluctuating interaction between local STIs, cytokine secretion and inflammation.

The effect of *N*. *gonorrhea* infection on HIV genital shedding at baseline corroborates previous reports associating this genital infection to increased local replication of HIV, local immune activation and cytokine secretion [7, 11, 12] and underscores the importance of treating genital infections in order to reduce the risk of sexual HIV transmission. The effect of baseline IL-8 on GVL at month 12 could relate to untreated yeast and trichomonas infections or to cervicitis, promoting HIV genital shedding at month 12 [43–45], whilst the effect of MIP-1β could be due to HIV-induced secretion of antimicrobial substances by immune cells.

The exploration of the association between intrinsic anti-viral factors, GVL and IFN-induced cytokines remains inconclusive. Although we report the possibility that that low level genital BST-2 expression might be associated with high GVL, neither cellular mRNA expression of BST2 nor APOBEC 3G correlated with soluble IP-10 in the genital tract. These findings could be related to first, the lack of direct link between levels of cell-associated BST2 and APOBEC3G and levels of extra-cellular HIV RNA and soluble IP-10, in clinical blood and genital samples. Secondly, restriction factors expressed in heterogeneous population of blood and mucosal cells might poorly correlate with VL, since only a fraction of the cells analyzed represent targets and source for HIV. Thirdly, despite the clear anti-viral properties of APOBEC3G and BST2 *in vitro*, their effect *in vivo*, might be detectable only at supra-physiological expression induced by supplementation of IFN-α [18].

Our work has several limitations. The follow-up period included only two time points at a year interval. The possible occurrence and consequences of blips of viral replication in the plasma and the genital tract at intermediate time points could not be investigated. Quite a number of samples were not available in sufficient volume to perform the whole panel of testing, thereby reducing the statistical power of the calculation. An important consequence might be that the frequency of HIV genital shedding episodes during suppressive ART might have been under-estimated in this study. Bacterial vaginosis and yeast infections, likely to be frequent in this setting were not assessed in this cohort. In addition, the treatment of STIs followed a syndromic approach since laboratory results were not available in real time, with the risk of missing asymptomatic women. Hence, an accurate triangulation between the presence of STI, elevated inflammation and HIV shedding in the genital tract could not be done. Finally, 8 of the 19 cytokines in the panel evaluated yielded very low concentrations, and could not be analyzed. The CVL sampling method based on vaginal douches using 10mL of PBS may have diluted some cytokines levels below the detection limit, possibly resulting in only a fragmentary determination of the mucosal inflammation at baseline and/or month 12.

In conclusion, the present set of data clearly indicates that although HIV genital shedding primarily depends on plasma viral load it is also influenced by other factors, including local concentration of certain cytokines. The failure of ART to alter cytokines levels, underscores the persistent risk of HIV genital shedding and HIV transmission among patients with undetectable plasma viral load. Further investigation assessing HIV genital shedding at closer intervals and in larger cohorts are warranted to further delineate the possible role of cytokines on the risk of HIV transmission during suppressive ART. The causative role of various STI or vaginal washing practices in promoting genital inflammation and GVL needs to be determined. These data could inform the design and assess the relative effectiveness of various approaches to reduce the odds of HIV transmission during suppressive ART in the context of the Rwandan HIV program.

## Supporting Information

S1 Dataset Information on age, treatment, plasma and cervico-vaginal viral load, CD4 count, levels of APOBEC 3G, BST2 and cytokines among all study participants at baseline and at month 12. (XLSX)

S2 Dataset Standard curves and raw data from the measurement of 19 cytokines in cervico-vaginal lavages of study participants at baseline and after 12 months. (XLS)
